# What is a predatory journal? A scoping review

**DOI:** 10.12688/f1000research.15256.2

**Published:** 2018-08-23

**Authors:** Kelly D. Cobey, Manoj M Lalu, Becky Skidmore, Nadera Ahmadzai, Agnes Grudniewicz, David Moher

**Affiliations:** 1 Centre for Journalology, Clinical Epidemiology Program, Ottawa Hospital Research Institute, Ottawa, K1H 8L6, Canada; 2 School of Epidemiology and Public Health, Faculty of Medicine, University of Ottawa, Ottawa, K1G 5Z3, Canada; 3 Department of Psychology, School of Natural Sciences, University of Stirling, Stirling, FK9 4LA, UK; 4 Department of Anesthesiology and Pain Medicine, The Ottawa Hospital, Faculty of Medicine, University of Ottawa; Regenerative Medicine Program, Ottawa Hospital Research Institute, Ottawa, K1H 8L6, Canada; 5 Telfer School of Management, University of Ottawa, Ottawa, K1N 6N5, Canada

**Keywords:** scholarly publishing, open access, predatory journals, predatory publishers, illegitimate journals, peer review, reporting quality

## Abstract

**Background:** There is no standardized definition of what a predatory journal is, nor have the characteristics of these journals been delineated or agreed upon. In order to study the phenomenon precisely a definition of predatory journals is needed. The objective of this scoping review is to summarize the literature on predatory journals, describe its epidemiological characteristics, and to extract empirical descriptions of potential characteristics of predatory journals.

**Methods:** We searched five bibliographic databases: Ovid MEDLINE, Embase Classic + Embase, ERIC, and PsycINFO, and Web of Science on January 2 ^nd^, 2018. A related grey literature search was conducted March 27 ^th^, 2018. Eligible studies were those published in English after 2012 that discuss predatory journals. Titles and abstracts of records obtained were screened. We extracted epidemiological characteristics from all search records discussing predatory journals. Subsequently, we extracted statements from the empirical studies describing empirically derived characteristics of predatory journals. These characteristics were then categorized and thematically grouped.

**Results:** 920 records were obtained from the search. 344 of these records met our inclusion criteria. The majority of these records took the form of commentaries, viewpoints, letters, or editorials (78.44%), and just 38 records were empirical studies that reported empirically derived characteristics of predatory journals. We extracted 109 unique characteristics from these 38 studies, which we subsequently thematically grouped into six categories: journal operations, article, editorial and peer review, communication, article processing charges, and dissemination, indexing and archiving, and five descriptors.

**Conclusions:** This work identified a corpus of potential characteristics of predatory journals. Limitations of the work include our restriction to English language articles, and the fact that the methodological quality of articles included in our extraction was not assessed. These results will be provided to attendees at a stakeholder meeting seeking to develop a standardized definition for what constitutes a predatory journal.

## Introduction

The term ‘predatory journal’ was coined less than a decade ago by Jeffrey Beall ^1^. Predatory journals have since become a hot topic in the scholarly publishing landscape. A substantial body of literature discussing the problems created by predatory journals, and potential solutions to stop the flow of manuscripts to these journals, has rapidly accumulated ^2– 6^. Despite increased attention in the literature and related educational campaigns ^7^, the number of predatory journals, and the number of articles these journals publish, continues to increase rapidly ^8^. Some researchers may be tricked into submitting to predatory journals ^9^, while others may do so dubiously to pad their curriculum vitae for career advancement ^10^.

One factor that may be contributing to the rise of predatory journals is that there is currently no agreed upon definition of what constitutes a predatory journal. The characteristics of predatory journals have not been delineated, standardized, nor broadly accepted. In the absence of a clear definition, it is difficult for stakeholders such as funders and research institutions to establish explicit policies to safeguard work they support from being submitted to and published in predatory journals. Likewise, if characteristics of predatory journals have not been delineated and accepted, it is difficult to take an evidence-based approach towards educating researchers on how to avoid them. Establishing a consensus definition has the potential to inform policy and to significantly strengthen educational initiatives such as Think, Check, Submit ^7^.

The challenge of defining predatory journals has been recognized ^11^, and recent discussion in the literature highlights a variety of potential definitions. Early definitions by Beall describe predatory publishers as outlets “which publish counterfeit journals to exploit the open-access model in which the author pays” and publishers that were “dishonest and lack transparency” ^1^. Others have since suggested that we move away from using the term ‘predatory journal’, in part because the term neglects to adequately capture journals that fail to meet expected professional publishing standards, but do not intentionally act deceptively ^12– 15^. This latter view suggests that the rise of so-called predatory journals is not strictly associated with dubious journal operations that use the open-access publishing model (e.g., publishing virtually anything to earn an article processing charge (APC)), but represents a wider spectrum of problems. For example, there is the conundrum that some journals hailing from the global south may not have the knowledge, resources, or infrastructure to meet best practices in publishing although some of them have ‘international’ or ‘global’ in their title. Devaluing or black-listing such journals may be problematic as they serve an important function in ensuring the dissemination of research on topics of regional significance.

Other terms to denote predatory journals such as “illegitimate journals ^9, 16^”, “deceptive journals ^15^”, “dark” journals ^17^, and “journals operating in bad faith ^13^” have appeared in the literature, but like the term “predatory journal” they are reductionist ^11^ and may not adequately reflect the varied spectrum of quality present in the scholarly publishing landscape and the distinction between low-quality and intentionally dubious journals. These terms have also not garnered widespread acceptance, and it is possible that the diversity in nomenclature leads to confusion for researchers and other stakeholders.

Here, we seek to address the question “what is a predatory journal?” by conducting a scoping review ^18, 19^ of the literature. Scoping reviews are a type of knowledge synthesis that follow a systematic approach to map the literature on a topic, and identify the main concepts, theories and sources, and determine potential gaps in that literature. Guidance on their conduct is available ^18– 20^ and guidance on their reporting is forthcoming. Our aims are twofold. Firstly, in an effort to provide an overview of the literature on the topic, we seek to describe epidemiological characteristics of all records discussing predatory journals. Secondly, we seek to synthesize the existing empirically derived characteristics of predatory journals. The impetus for this work is to establish a list of evidence-based traits and characteristics of predatory journals. This corpus of possible characteristics of predatory journals is one source that could be considered by an international stakeholders meeting to generate a consensus definition of predatory journals. Other sources will be included (e.g., ^8^).

## Methods

### Transparency statement

Prior to initiating this study, we drafted a protocol that was posted on the Open Science Framework prior to data analysis (please see: https://osf.io/gfmwr/). We did not register our review with PROSPERO as the registry does not accept scoping reviews. Other than the protocol deviations described below, the authors affirm that this manuscript is an honest, accurate, and transparent account of the study being reported; that no important aspects of the study have been omitted; and that discrepancies from the study as planned have been explained. We briefly re-state our study methods here. Large sections of the methods described here are taken directly from the original protocol. We used the PRISMA statement ^21^ to guide our reporting of this scoping review.

### Search strategy

For our full search strategy please see Supplementary File 1. An experienced medical information specialist (BS) developed and tested the search strategy using an iterative process in consultation with the review team. Another senior information specialist peer reviewed the strategy prior to execution using the PRESS Checklist ^22^. We searched a range of databases in order to achieve cross-disciplinary coverage. These included: Web of Science and four Ovid databases: Ovid MEDLINE®, including Epub Ahead of Print and In-Process & Other Non-Indexed Citations, Embase Classic + Embase, ERIC, and PsycINFO. We performed all searches on January 2, 2018.

There were no suitable controlled vocabulary terms for this topic in any of the databases. We used various free-text phrases to search, including multiple variations of root words related to publishing (e.g., edit, journal, publication) and predatory practices (e.g., bogus, exploit, sham). We adjusted vocabulary and syntax across the databases. We limited results to the publication years 2012 to the present, since 2012 is the year in which the term “predatory journal” reached the mainstream literature ^1^.

We also searched abstracts of relevant conferences (e.g., The Lancet series and conference “Increasing Value, Reducing Waste”, International Congresses on Peer Review and Scientific Publication) and Google Scholar to identify grey literature. For the purposes of our Google Scholar search, we conducted an advanced search (on March 27, 2018) using the keywords: predatory, journal, and publisher. We restricted this search to content published from 2012 onward. A single reviewer (KDC) reviewed the first 100 hits and extracted all potentially relevant literature encountered for review, based on title. We did not review content from file sources that were from mainstream publishers (e.g., Sage, BMJ, Wiley), as we expected these to be captured in our broader search strategy.

### Study population and eligibility criteria

Our study population included articles, reports, and other digital documents that discuss, characterize, or describe predatory journals. We included all study designs from any discipline captured by our search that were reported in English. This included experimental and observational research, as well as commentaries, editorials and narrative summaries in our epidemiological extraction. For extraction of characteristics of predatory journals we restricted our sample to studies that specifically provided empirically derived characteristics of predatory journals.

### Screening and data extraction

Data extraction forms were developed and piloted prior to data extraction. Details of the forms used are provided in the Open Science Framework, see here: https://osf.io/p5y2k/. We first screened titles and abstracts against the inclusion criteria. We verified full-text articles met the inclusion criteria and we extracted information on corresponding author name, corresponding author country, year of publication (we selected the most recent date stated), study design (as assessed by the reviewers), and journal name. We also extracted whether or not the paper provided a definition of a predatory journal. This was coded as yes/no and included both explicit definitions (e.g. “Predatory journals are…”) as well as implicit definitions.

When extracting data, we restricted our sample of articles to those that provided a definition of predatory journals, or described characteristics of predatory journals, based on empirical work (i.e., not opinion, not definitions which referenced previous work). Specifically, we restricted our sample of articles to those classed as having an empirical study design and then re-vetted each article to ensure that the study addressed defining predatory journals or their characteristics. For those articles included, we extracted sections of text statements describing the traits/characteristics of predatory journals. Extraction was done by a single reviewer, with verification conducted by a second reviewer. Conflicts were resolved via consensus. In instances where an empirically derived trait/characteristic of predatory journals was mentioned in several sections of the article, we extracted only a single representative statement.

### Data analysis

Our data analysis involved both quantitative (i.e., frequencies and percentages) and qualitative (i.e., thematic analysis) methods. First, a list of potential characteristics of predatory journals was generated collaboratively by the two reviewers who conducted data extraction (KDC, NA). Subsequently, each of the statements describing characteristics of predatory journals that were extracted from the included articles were categorized using the list generated. During the categorization of the extracted statements, if a statement did not apply to a category already on the list, a new category was added. Where duplicate statements were inadvertently extracted from a single record we categorized these only once. During the categorization and grouping process, details on the specific wording of statements from specific included records were not retained (i.e., our categories and our themes do not preserve the original wording of the extracted text).

Subsequently, in line with Galipeau and colleagues ^23^, after this initial categorization, we collated overlapping or duplicate categories into themes. Then, two reviewers (KDC, AG) evaluated recurring themes in the work to synthesize the data. A coding framework was iteratively developed by KDC and AG by coding each characteristic statement independently and inductively (i.e., without using a theory or framework a priori). The two reviewers met to discuss these codes, and through consensus decided on the final themes and their definitions. The reviewers then went back to the data and recoded with the agreed-upon themes. Lastly, the reviewers met to compare assignment of themes to statements. Discrepancies were resolved by consensus. Two types of themes emerged: *categories* (i.e., features of predatory journals to which the statements referred) and *descriptors* (i.e., statements which described these features, usually with either a positive or negative value).

### Deviations from study protocol

We conducted data extraction of epidemiological characteristics of papers discussing predatory journals in duplicate. The original protocol indicated this would be done by a single reviewer with verification. The original protocol stated we would extract information on the discipline of the journals publishing our articles included for epidemiological data extraction (as defined by MEDLINE). Instead, we used SCIMAGOJR (SJR) ( https://www.scimagojr.com/) to determine journal subject areas post-hoc and only extracted this information for the included empirical articles describing empirically derived characteristics of predatory journals. For included articles, post-hoc, we decided to extract information on whether or not the record reported on funding.

## Results

### Search results and epidemiological characteristics

**Figure 1.**
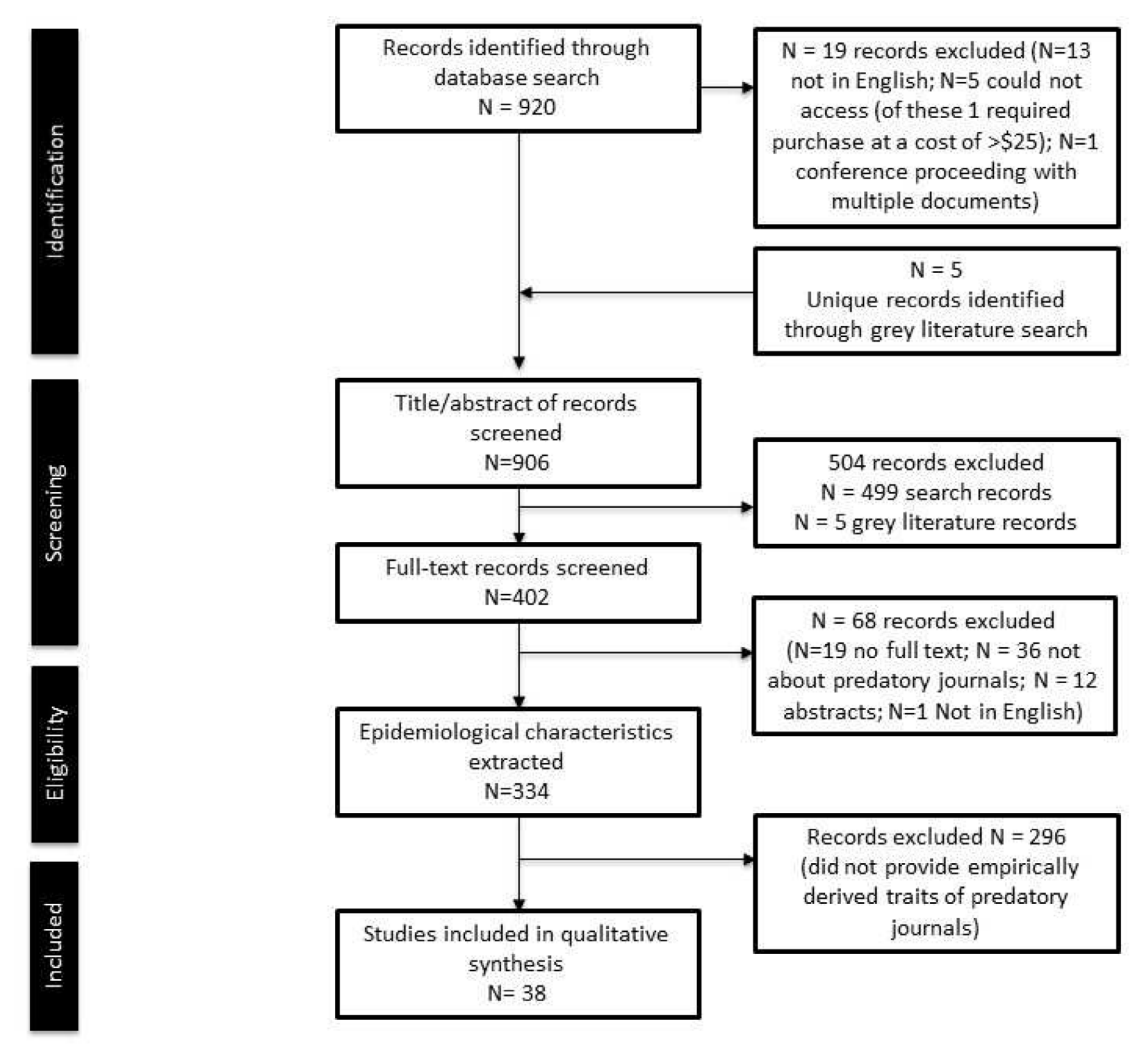
Preferred Reporting Items for Systematic reviews and Meta-Analyses (PRISMA) flow diagram summarizing study selection.

Please see Figure 1 for record and article flow during the review. The original search captured 920 records. We excluded 19 records from initial screening because they were not in English (N = 13), we could not access a full-text document (N = 5; of which one was behind a paywall at a cost of greater than $25 CAD), or the reference referred to a conference proceeding containing multiple documents (N = 1).

We screened a total of 901 title and abstract records obtained from the search strategy. Of these, 402 were included for full-text screening. 499 records were excluded for not meeting our study inclusion criteria. After full-text screening of the 402 studies, 334 were determined to have full texts and to discuss predatory journals. The remaining 68 records were excluded because: they were not about predatory journals (N = 36), did not have full texts (N = 19), were abstracts (N = 12), or were published in a language other than English (N = 1). The 334 articles included for epidemiological data extraction were published between 2012 and 2018 with corresponding authors from 43 countries. The number of publications mentioning predatory journals increased each year from 2012 to 2017 (See Table 1). The vast majority of these publications took the form of commentaries, viewpoints, letters, or editorials (262/334; 78.44%).

**Table 1.** Epidemiological characteristics of all articles mentioning predatory journals and those included empirical articles describing empirically derived characteristics of predatory journals included in our scoping review.

	Articles mentioning predatory journals (N=334)	Empirical articles included in systematic scoping review (N=38)
**Nationality of** **corresponding** **authors (Top 3)**	USA: 78 India: 34 Canada: 22 ^i^	USA: 11 Italy: 5 Canada: 4 ^ii^
**Publication** **year of** **articles** ^iii^	2012: 5 2013: 8 2014: 22 2015: 71 2016: 78 2017:140 2018: 5 Not reported: 5	2012: 0 2013: 0 2014: 2 2015: 9 2016: 10 2017: 16 2018: 1 Not reported: 0
**Study design**	Commentary/Viewpoint/Editorial/Letter: 262 Observational Study: 34 Narrative Review: 20 Case report/Case series: 13 Systematic Review: 1 Other: 4	Commentary/Viewpoint/Editorial/Letter: 0 Observational Study: 26 Narrative Review: 0 Case report/Case series: 11 Systematic Review: 1 Other: 0

^i^ 61 articles did not clearly state the corresponding authors’ nationality, and 1 stated they wished to remain anonymous ^ii^ 1 article did not clearly state the corresponding author’s nationality ^iii^ Note this is truncated data for 2018 since we conducted out search on January 2nd, 2018

Of the articles discussing predatory journals, only 38 specifically described a study that reported empirically derived characteristics or traits of predatory journals. These studies were published between 2014 and 2018 and produced by corresponding authors from 19 countries. The majority of these included studies were observational studies (26/38; 68.4%) (See Table 1 and Table 2).

**Table 2.** Included empirical records (N=38). For full citations see Supplementary File 2.

RefId	Corresponding author	County of corresponding author	Year of publication	Journal Title	Subject Area (from SJR)	Study design	Number of extracted characteristics (N=350)
**1**	Marilyn H. Oermann	USA	2017	Nursing Outlook	Nursing	Observational	14
**8**	Terence V. McCann	Australia	2017	Journal of Advanced Nursing.	Nursing	Systematic Review	10
**13**	Eric Mercier	Canada	2017	Postgraduate Medical Journal	Medicine	Observational	14
**35**	Pravin Bolshete	India	2018	Current Medical Research and Opinion	Medicine	Case report/ Case Series	13
**99**	Franca Deriu	Italy	2017	Neuroscience	Neuroscience	Observational	8
**121**	Mary M. Christopher	USA	2015	Frontiers in Veterinary Science	N/A	Observational	34
**150**	Marilyn H. Oermann	USA	2016	Journal of Nursing Scholarship	Nursing	Observational	14
**165**	Katarzyna Pisanski	UK	2017	Nature	Multidisciplinary	Observational	8
**168**	Andrea Manca	Italy	2017	Archives of Physical Medicine and Rehabilitation	Health Professions/ Medicine	Observational	9
**176**	Bhakti Hansoti	USA	2016	Western Journal of Emergency Medicine	Medicine	Observational	2
**181**	Victor Grech	Malta	2016	Journal of Visual Communication in Medicine	Arts and Humanities/ Health Professions	Case report/ Case Series	5
**203**	Jelte M. Wicherts	The Netherlands	2016	PLOS ONE	Agriculture and Biological Sciences/ Biochemistry, Genetics and Molecular Biology; Medicine	Observational	1
**209**	Cenyu Shen	Finland	2015	BMC Medicine	Medicine	Observational	6
**275**	Dragan Djuric	Serbia	2015	Science and Engineering Ethics	Buisness, Management and Accounting; Medicine; Nursing; Social Sciences	Case report/ Case Series	5
**299**	Larissa Shamseer	Canada	2017	BMC Medicine	Medicine	Observational	27
**362**	Mark Clemons	Canada	2017	The Oncologist	N/A	Case report/ Case Series	15
**384**	David Moher	Canada	2015	BMC Medicine	Medicine	Case report/ Case Series	11
**462**	Lynn E. McCutcheon	USA	2016	North American Journal of Psychology	Psychology; Social Sciences	Observational	6
**489**	Anonymous	Anonymous	2015	Journal of Developmental & Behavioral Pediatrics	Medicine; Psychology	Case report/ Case Series	12
**525**	Tove Faber Frandsen	Denmark	2017	Scientometrics	Computer Science; Social Science	Observational	1
**548**	Jaimie A. Teixeira Da Silva	Japan	2017	Current Science	Multidisciplinary	Case report/ Case Series	6
**561**	P. de Jager	South Africa	2017	South African Journal of Business Management	Business, Management and Accounting	Observational	13
**586**	Krystal E. Noga-Styron	USA	2017	Journal of Criminal Justice Education	Social Science	Observational	9
**596**	John H. McCool	USA	2017	The Scientist Magazine	N/A	Case report/ Case Series	4
**654**	Filippo Eros Pani	Italy	2017	Library Review	Social Science	Observational	2
**660**	Marco Cosentino	Italy	2017	Plagiarism Across Europe and Beyond 2017- Conference Proceedings	N/A	Case report/ Case Series	2
**686**	Andrea Marchitelli	Italy	2017	Italian Journal of Library, Archives & Information Science	N/A	Observational	1
**701**	G. S. Seethapathy	Norway	2016	Current Science	Multidisciplinary	Observational	3
**728**	Alexandre Martin	USA	2016	Learned Publishing	Social Sciences	Case report/ Case Series	4
**736**	Marta Somoza- Fernández	Spain	2016	El profesional de la información	Computer Science; Social Sciences	Observational	6
**755**	Marcin Kozak	Poland	2016	Journal of the Association for Information Science and Technology	Computer Science; Decision Sciences; Social Sciences	Case report/ Case Series	19
**812**	Alexandru-Ionuţ Petrişor	Romania	2016	Malaysian Journal of Library & Information Science	Social Sciences	Observational	23
**900**	Jingfeng Xia	USA	2015	Journal of the Association for Information Science and Technology	Computer Science; Decision Sciences; Social Sciences	Observational	3
**904**	Mehrdad Jalalian	Iran	2015	Geographica Pannonica	Business, Managements and Accounting; Earth and Planetary Sciences; Social Sciences	Observational	8
**975**	Williams Ezinwa Nwagwu	South Africa	2015	Learned Publishing	Social Sciences	Observational	11
**976**	Jingfeng Xia	USA	2015	Learned Publishing	Social Sciences	Observational	6
**1012**	Ayokunle Olumuyiwa Omobowale	Nigeria	2014	Current Sociology	Social Sciences	Observational	5
**1068**	David Matthew Markowitz	USA	2014	121st ASEE Annual Conference & Exposition	N/A	Observational	10

Five additional records obtained from the grey literature search were excluded. These records were either duplicates of studies captured in the main search or they did not provide empirically derived characteristics of predatory journals.

### Mapping the data into emergent themes

The list generated to categorize the extracted statements describing characteristics of predatory journals had 109 categories. Two types of themes were identified using qualitative thematic analysis: categories and descriptors. Each statement addressed at least one of the following categories: journal operations, article, editorial and peer review, communication, article processing charges, and dissemination, indexing, and archiving. Within these categories, statements used descriptors including: deceptive or lacking transparency, unethical research or publication practices, persuasive language (), poor quality standards, or high quality standards. Statements that did not include a descriptive component (i.e., were neutral) were coded as not applicable (See Table 3 for themes and definitions). Statements addressing more than one category or using more than one descriptor were coded multiple times. Below we briefly summarize the qualitative findings by category (For full results, see Table 4).

**Table 3.** Themes and Definitions used to Code Characteristics of Predatory Journals.

Theme	Definition
**Category**	
** 1. Journal Operations**	Features related to how the journal conducts its business operations
** 2. Article**	Features related to articles appearing in the journal
** 3. Editorial and Peer Review**	Any aspect of the internal or external review of submitted articles and decisions on what to publish
** 4. Communication**	How the journal interacts with (potential) authors, editors, and readers
** 5. Article Processing Charges**	Fees taken in by journal as part of their business model
** 6. Dissemination, Indexing, and Archiving**	Information on how the journal disseminates articles and use of indexing and archiving tools
**Descriptor**	
** 1. Deceptive or Lacking Transparency**	Intentionally deceitful practice; Practices or processes that are not made clear to the reader; Missing information
** 2. Unethical Research or Publication Practices**	Violations of accepted publication and research ethics standards (e.g., Committee on Publication Ethics guidelines)
** 3. Persuasive Language**	Language that targets; Language that attempts to convince the author to do or believe something
** 4. Poor Quality Standards**	Lack of rigour in journal operations; Lack of professional standards/ practices; missing information; Poor quality writing or presentation (e.g., grammatical or spelling errors)
** 5. High Quality Standards**	Evidence of rigour in journal operations; Evidence that professional standards/practices are being met; Clear information
** 6. Not Applicable**	Neutral or non-descriptive statement

**Table 4.** Characteristics extracted, including article reference and frequency, and their thematic categorization and descriptor.

Characteristics	Frequency	RefIDs	Category	Descriptor
**Article authors not listed with credentials/contact info**	1	1	Article	Poor Quality Standards; Deceptive or Lacking Transparency
**Articles follow Introduction, Methods, Results, and** **Discussion (IMRaD) structure for reporting**	1	1	Article	NA
**Articles have logical presentation and organization**	1	1	Article	High Quality Standards
**Items expected to be reported were reported most of the** **time (e.g. research question, sampling procedure)**	1	1	Article	High Quality Standards
**Many studies failed to report REB/ethics approval**	1	1	Article	Unethical Research or Publication Practices
**Wide range of lengths of articles**	1	1	Article	NA
**Wide range of reference styles used**	1	1	Article	Poor Quality Standards
**Articles contain statistical and methods errors**	1	462	Article	Poor Quality Standards
**Journals contain articles with plagiarized content**	3	1, 121, 275	Article	Unethical Research or Publication Practices; Deceptive or Lacking Transparency
**Grammatical errors in articles**	4	1, 121, 462, 561	Article	Poor Quality Standards
**Quality of articles rated as poor**	5	1,8, 121, 462, 1012	Article	Poor Quality Standards
**Articles are poorly cited**	5	525, 561, 654 900, 975	Article	Poor Quality Standards
**Journal offers discounts on the standard open access** **charges e.g. for specific members, a fee waiver to authors** **from low-income economies**	1	812	Article Processing Charges	NA
**Journals highlight easy methods of payment e.g. PayPal,** **credit card, debit card, net banking, cash card online 24/7.**	1	812	Article Processing Charges	Persuasive Language
**Journal APCs clearly stated**	4	561, 755, 812, 976	Article Processing Charges	High Quality Standards
**Journal does not specify APCs**	9	35, 99, 121, 150, 181, 299, 489, 755, 976	Article Processing Charges	Deceptive or Lacking Transparency
**Journal has hidden APCs or hidden information on APCs**	9	121, 150, 168, 181, 299, 362, 489, 586, 976	Article Processing Charges	Deceptive or Lacking Transparency; Poor Quality Standards; Unethical Research or Publication Practices
**APCs are lower than at legitimate journals**	9	99, 168, 181, 209, 299, 362, 561, 812, 976	Article Processing Charges	NA
**E-mail invitations explicitly noted they were not spam**	1	13	Communication	Persuasive Language
**E-mail solicitations don't contain contact information**	1	13	Communication	Poor Quality Standards; Deceptive or Lacking Transparency
**E-mail solicitations note acceptance of all manuscript** **types**	1	13	Communication	Persuasive Language
**Journal preys on junior researchers**	1	121	Communication	Deceptive or Lacking Transparency
**Journal language targets authors**	1	299	Communication	Persuasive Language
**E-mail invitations addressed inappropriately**	1	384	Communication	Poor Quality Standards
**Journals use the same strategies as internet-based scams** **to identify their prey**	1	812	Communication	Deceptive or Lacking Transparency
**Journal or e-mail invitations stress ability to publish in a** **special issue**	2	13, 35	Communication	Persuasive Language
**E-mails invitations specified a deadline to submit**	2	13, 362	Communication	Persuasive Language
**E-mail invitations had an unsubscribe option**	2	13, 384	Communication	High Quality standards
**E-mail solicitations have grammar errors**	2	13, 384	Communication	Poor Quality Standards
**E-mail solicitations referenced researchers previous work**	2	13, 384	Communication	Persuasive Language
**Journal uses business advertisement terminology**	2	561, 812	Communication	Persuasive Language
**Journals use positive emotions, linguistic qualifiers, or** **few casual words to accomplish their goal of selling the** **publication**	2	975, 1068	Communication	Persuasive Language
**Journals solicit editors via aggressive e-mail tactics**	3	8, 13, 586	Communication	Persuasive Language
**E-mail solicitations are not relevant to researcher** **expertise**	3	362, 384, 755	Communication	Poor Quality Standards
**E-mail invites were overly formal or used praise**	3	362, 384, 812	Communication	Persuasive language
**Journals solicit papers via aggressive e-mail tactics**	13	8, 13, 121, 150, 181, 362, 384, 489, 548, 586, 596, 755, 904	Communication	Persuasive Language
**E-mail solicitations don't mention APCs**	3	13, 489, 586	Communication; Article Processing Charges	Deceptive or Lacking Transparency
**E-mail solicitations or journal note special discounts**	4	13, 755, 812, 904	Communication; Article Processing Charges	Persuasive Language
**Journals contain extreme variability in article quality**	1	462	Dissemination, Indexing, Archiving	Poor Quality Standards
**Journal notes fake abstracting and indexing**	1	812	Dissemination, Indexing, Archiving	NA
**Journals can be found on Google Scholar**	1	975	Dissemination, Indexing, Archiving	NA
**Journals have Digital Object Identifiers (DOIs)**	1	975	Dissemination, Indexing, Archiving	High Quality Standards
**Journals are not archived**	2	8, 150	Dissemination, Indexing, Archiving	Poor Quality Standards
**Journals tend not to mention Committee of Publication** **Ethics (COPE)**	2	299, 384	Dissemination, Indexing, Archiving	Poor Quality Standards
**Journals state they are open access but are not openly** **available**	2	362, 755	Dissemination, Indexing, Archiving	Unethical Research or Publication Practices; Deceptive or Lacking Transparency
**Journals have a subscription based model**	2	755, 561	Dissemination, indexing, archiving	NA
**Journals may have International Standard Serial Number** **(ISSN)**	3	701, 736, 975	Dissemination, Indexing, Archiving	NA
**Articles may be in PubMed**	4	99, 150, 168, 654	Dissemination, Indexing, Archiving	NA
**Journals may not be in the Directory of Open Access** **Journals**	5	35, 99, 561, 755, 975	Dissemination, Indexing, Archiving	Poor Quality Standards
**Journals are not indexed**	7	8,121,150,181,561, 736, 975	Dissemination, Indexing, Archiving	Poor Quality Standards
**Journal may be listed in Directory of Open Access** **Journals**	8	168, 209, 299, 561, 686, 736, 755, 975	Dissemination, Indexing, Archiving	NA
**Journals state they are open access**	11	8, 13, 99, 165, 168,176, 181, 209, 299, 362, 755	Dissemination, Indexing, Archiving	NA
**Journals entice big name scientists to lend name (only) to** **editorial board**	1	121	Editorial/Peer Review	Persuasive Language; Unethical Research or Publication Practices
**Journal describes peer review process clearly**	1	150	Editorial/Peer Review	High Quality Standards
**Journal conducts fake reviews or editorial review**	2	121, 165	Editorial/Peer Review	Deceptive or Lacking Transparency; Poor Quality Standards; Unethical Research or Publication Practices
**Editorial board has an agenda to publish certain articles** **(from certain authors)**	2	121, 561	Editorial/Peer Review	Deceptive or Lacking Transparency; Unethical Research or Publication Practices
**Journals have poor editorial oversight/review**	2	462, 755	Editorial/Peer Review	Poor Quality Standards
**Editorial board repeats in multiple journals**	3	35, 362, 812	Editorial/Peer Review	Unethical Research or Publication Practices
**Journal conducts peer review**	6	150, 299, 362, 384, 489, 586	Editorial/Peer Review	High Quality Standards
**Editorial board is not stated or incomplete**	7	35, 150, 299, 548, 755, 812, 1068	Editorial/Peer Review	Deceptive or Lacking Transparency
**Journals have short peer review times**	7	548, 586, 596, 728, 755, 812, 1068	Editorial/Peer Review	NA
**Journals conduct poor quality peer review**	8	8, 121,165,489, 596, 728,1012,1068	Editorial/Peer Review	Poor Quality Standards
**Authors more likely to come from second-tier academic** **institutes**	1	701	Editorial/Peer Review; Article	NA
**Editor inserts plagiarized content into article**	1	728	Editorial/Peer Review; Article	Deceptive or Lacking Transparency; Unethical Research or Publication Practices
**Editors or websites listed on page may not even be** **affiliated to journal**	1	121	Editorial/Peer Review; Journal Operations	Deceptive or Lacking Transparency
**Journal does not look at all submitting authors fairly**	1	121	Editorial/Peer Review; Journal Operations	Deceptive or Lacking Transparency; Unethical Research or Publication Practices
**Journal requests fees to sit on editorial board**	1	165	Editorial/Peer Review; Journal Operations	Poor Quality Standards; Unethical Research or Publication Practices
**Editorial board lacks legitimacy (appointed without** **knowledge, wrong skillset)**	7	121, 150, 165, 299, 489, 812, 1068	Editorial/Peer Review; Journal Operations	Deceptive or Lacking Transparency; Poor Quality Standards; Unethical Research or Publication Practices
**Journal has no article preparation instructions**	1	35	Journal Operations	Poor Quality Standards
**A journal that collects information for less-than-honorable** **purposes**	1	121	Journal Operations	Deceptive or Lacking Transparency
**A journal that commercially encroaches on existing** **journals**	1	121	Journal Operations	Deceptive or Lacking Transparency
**A journal that seeks to discredit another journal**	1	121	Journal Operations	Deceptive or Lacking Transparency
**Journal actively seeks manuscripts to prevent other** **journals from publishing**	1	121	Journal Operations	Deceptive or Lacking Transparency; Unethical Research or Publication Practices
**Journal shuts down ideas and results of submitted** **articles**	1	121	Journal Operations	NA
**Journal will publish non-academic research**	1	121	Journal Operations	Poor Quality Standards; Unethical Research or Publication Practices
**Grammatical errors on journal webpage**	1	299	Journal Operations	Poor Quality Standards
**Journal uses distorted or unauthorized images**	1	299	Journal Operations	Deceptive or Lacking Transparency; Unethical Research or Publication practices
**Journals indicate they retain copyright in spite of stating** **journal was OA**	1	299	Journal Operations	Unethical research or publication practices; Deceptive or Lacking Transparency
**Journal contains articles that should be combined into** **one (e.g., salami publishing)**	1	561	Journal Operations	Unethical Research or Publication Practices
**Journals tend to publish in high quantity without regard** **for quality to earn profit**	1	561	Journal Operations	Unethical Research or Publication Practices; Poor Quality Standards
**Journals may publish work funded by national** **governments**	1	701	Journal Operations	NA
**Journal names change with trends**	1	812	Journal Operations	Poor Quality Standards
**Journal is not very readable**	1	1068	Journal Operations	Poor Quality Standards
**Journal has no authorship policy**	2	35, 121	Journal Operations	Unethical Research or Publication Practices
**Journal offer authors incentives to publish**	2	121, 165	Journal Operations	Persuasive Language
**Article submission occurs via email**	2	35, 299	Journal Operations	Poor Quality Standards
**Journal has hidden publishing contract information**	2	121, 362	Journal Operations	Deceptive or Lacking Transparency
**Journals have the goal to make money without regard for** **quality**	2	121, 462	Journal Operations	Deceptive or Lacking Transparency; Poor Quality Standards
**Journals solicit papers under false pretenses**	2	121, 489	Journal Operations	Deceptive or Lacking Transparency
**Journal contains duplicate publications**	2	121, 561	Journal Operations	Unethical Research or Publication Practices
**Journals do not mention reporting guidelines**	2	299, 384	Journal Operations	Poor Quality Standards
**Journals tend not to have legitimate impact factor**	2	299, 904	Journal Operations	Deceptive or Lacking Transparency
**Description of the manuscript handling process is lacking**	2	299, 1012	Journal Operations	Deceptive or Lacking Transparency; Poor Quality Standards
**Journal has no plagiarism policy/duplicate publication** **policy**	3	35, 121, 299	Journal Operations	Unethical Research or Publication Practices
**Journals do not have retraction/correction policies**	3	299, 489, 660	Journal Operations	Unethical research or publication practices
**Journal publishes studies without authors’ agreement**	4	121, 150, 489, 660	Journal Operations	Unethical Research or Publication Practices
**Journal names specify 'worldly' or 'global' nature of** **journal**	4	275, 362, 812, 1068	Journal Operations	Persuasive Language
**Journals contact information is not professional (e.g.,** **Gmail accounts)**	4	299, 812, 904, 1068	Journal Operations	Poor Quality Standards
**Journals display fake metrics**	4	13,275,299,812	Journal Operations	Deceptive or Lacking Transparency
**Present content unrelated to the journal readership/scope/** **journal title**	5	1, 121, 150, 275, 299	Journal Operations	Poor Quality Standards
**Journal contains broken links/domain for sale**	5	35, 168, 299, 586, 755	Journal Operations	Poor Quality Standards
**Journals have short/rapid publication times**	7	150, 548,586,596, 812, 975, 1068	Journal Operations	NA
**Journals do not contain any articles**	8	1, 35, 99, 168, 209, 299, 548, 586, 755	Journal Operations	Poor Quality Standards
**Journal lists few articles**	8	35, 99, 150, 168, 362, 900, 975, 976	Journal Operations	NA
**Journals closely copy/plagiarize names or websites of** **legitimate journals/publishers**	8	1,18,165,299,548,736,812,904	Journal Operations	Deceptive or Lacking Transparency
**Journals display deceptive information or misleading** **claims about their practices**	8	121,165,489,736,755,812,904, 1068	Journal Operations	Deceptive or Lacking Transparency
**Contact details of publisher absent or not easily verified**	11	35, 99, 121, 168, 209, 299, 362, 489, 755, 812, 904	Journal Operations	Deceptive or Lacking Transparency
**Journals display low levels of transparency, integrity, poor** **quality practices of journal operations**	14	8, 121, 203, 275, 299, 362, 384, 728, 736, 755, 812, 904, 1012, 1068	Journal Operations	Poor Quality Standards; Deceptive or Lacking Transparency
**Journals are published by/in predominantly by authors** **from specific countries**	10	1,8, 121, 209, 299, 755, 812, 900, 975, 976	Journal Operations; Article	NA

***Journal Operations.*** Predatory journal operations were described as: being deceptive or lacking transparency (19 statements), demonstrating poor quality standards (17 statements), demonstrating unethical research or publication practices (14 statements), using persuasive language (two statements). Five statements were neutral or non-descriptive. The most common characteristics of the journal operations category were “Journals display low levels of transparency, integrity, poor quality practices of journal operations” (N=14 articles); “Contact details of publisher absent or not easily verified” (N=11 articles); and “Journals are published by/in predominantly by authors from specific countries” (N=10 articles).

***Article.*** Articles in predatory journals were described as: demonstrating poor quality standards (six statements), demonstrating high quality standards (two statements), being deceptive or lacking transparency (three statements), and demonstrating unethical research of publication practices (three statements). Four statements were neutral or non-descriptive. The most common characteristics of the article category were: “Journals are published by/in predominantly by authors from specific countries” (N=10 articles); “Quality of articles rated as poor” (N=5 articles); and “Articles are poorly cited” (N=5 articles).

***Editorial and Peer Review.*** The editorial and peer review process was described as: demonstrating unethical or research practices (eight statements), being deceptive or lacking transparency (seven statements), demonstrating poor quality standards (five statements), demonstrating high quality standards (two statements), and using persuasive language (one statement). Two statements were neutral or non-descriptive. The most common characteristics of the editorial and peer review category were: “Journals conduct poor quality peer review” (N=8 articles) and “Journals have short peer review times”; “Editorial board is not stated or incomplete”; “Editorial broad lacks legitimacy (appointed without knowledge, wrong skillset)” (N=7 articles each).

***Communication.*** Communication by predatory journals was described as: using persuasive language (12 statements), demonstrating poor quality standards (four statements), being deceptive or lacking transparency (four statements), and demonstrating high quality standards (one statement). All communication statements were descriptive. The most common characteristic of the communications category was: “Journals solicit papers via aggressive e-mail tactics” (N=13 articles).

***Article Processing Charges.*** Article processing charges in predatory journals were described as: being deceptive or lacking transparency (three statements), using persuasive language (two statements), demonstrating poor quality standards (one statement), demonstrating unethical research or publication practices (one statement), and demonstrating high quality standards (one statement). Two statements were neutral or non-descriptive. The most common characteristics of the article processing charges category were: “APCs are lower than at legitimate journals”; “Journal does not specify APCs”; and “Journal has hidden APCs or hidden information on APCs” (N=9 articles each).

***Dissemination, Indexing, and Archiving.*** Dissemination, indexing, and archiving were described as: demonstrating poor quality standards (five statements), demonstrating unethical research or publication practices (one statement), and as being deceptive or lacking transparency (one statement). Seven statements were neutral or non-descriptive. The most common characteristics of the dissemination, indexing, and archiving category were: “Journals state they are open access” (N=11 articles); “Journal may be listed in DOAJ” (N=8 articles); and “Journals are not indexed” (N=7 articles).

## Discussion

This scoping review identified 334 articles mentioning predatory journals, with corresponding authors from more than 40 countries. The trajectory of articles on this topic is increasing rapidly. As an example, our search captured five articles from 2012 and 140 articles from 2017. The majority of articles captured took the form of a commentary, editorial or letter; just 38 had relevant empirically derived characteristics of predatory journals. One possibility for why there is little empirical work on this topic may be that most funding agencies have not set aside funding for journalology or a related field of enquiry–research on research. There are recent exceptions to this ^24^, but in general such funds are not widely available. Of the 38 studies from which we extracted data, post-hoc we examined the percentage that reported funding, and found that just 13.16% (5/38) did, 21.05% (8/38) did not, and 65.79% (25/38) did not report information on funding. Even among the five studies that reported funding, several of these were not project funding specific to the research, but rather broader university chair or fellowship support.

A total of 109 unique characteristics were extracted from the 38 empirical articles. When examining these unique characteristics some clear contrasts emerge. For example, we extracted the characteristic “Journal APCs clearly stated” (N = 4 articles) as well as the characteristics “Journal does not specify APCs” (N = 9 articles) and “Journal has hidden APCs or hidden information on APCs” (N = 9 articles). Potential inconsistencies of the importance of epidemiological characteristics will make it difficult to define predatory journals. Without a (consensus) definition it will be difficult to study the construct in a meaningful manner. It also makes policy initiatives and educational outreach imprecise and potentially less effective.

We believe a cogent next move is to invite a broad spectrum of stakeholders to a summit. Possible objectives could be to develop a consensus definition of a predatory journal, discuss how best to examine the longitudinal impact of predatory journals, and develop collaborative policy and educational outreach to minimize the impact of predatory publishers on the research community. As a starting point for defining predatory journals, those involved in a global stakeholder meeting to establish a definition for predatory journals may wish to exclude all characteristics that are common to legitimate journals. Further, one could exclude all characteristics that are conflicting, or which directly oppose one another. Another fruitful approach may be to focus on characteristics that can easily be audited to determine if journals do or do not meet the expected standards.

The unique characteristics we extracted were thematically grouped into six categories and five descriptors. Although we did identify one positive descriptor, high quality standards, the majority of descriptors were negative. Most categories (all but ‘Communication’) also included neutral or non-descriptive statements. The presence of both positive and neutral descriptors points to an overlap between characteristics that describe predatory journals and those that are viewed as ‘legitimate’, further emphasizing the challenges in defining predatory journals. The category with the most statements was ‘Journal Operations’ with 19 statements describing operations as deceptive or lacking transparency. The ‘Communication’ category had the most statements described as persuasive (11 statements), highlighting the targeted language predatory journals may use to convince the reader toward a certain action. Unethical or unprofessional publication practices described statements in all but the ‘Communication’ category and were most frequent in ‘Journal Operations’ and ‘Editorial and Peer Review’. These findings point to issues of great concern in research and publishing and an urgency to develop interventions and education to protect researchers, funders, and knowledge users.

There are a number of relevant limitations of this work that should be acknowledged. Firstly, while we endeavoured to ensure our systematic search and grey literature appraisal was comprehensive, it is possible that we missed some relevant documents that would have contributed additional empirically derived characteristics of predatory journals. As an example, several authors of this manuscript recently published a paper containing relevant empirical data and predatory characteristics ^2^; however, because this work was published in a commentary format, which did not include an abstract or use the search terms in the article title, it was not picked up in our search. Indeed, part of the challenge of systematically searching on this topic is the lack of agreement and diversity of terms used to describe predatory journals. Further, reviewers deciding which articles to include based on our inclusion criteria had to make judgements on study designs and methods used. Due to inconsistent reporting and terminology, this was not always straightforward and may have resulted in inadvertent exclusions. Secondly, in keeping with accepted scoping review methodology, we did not appraise the methodological quality of the articles that were included in our extraction. This means that the characteristics extracted have not been considered in context to the study design or methodological rigour of the work. In addition, we only extracted definitions from empirical studies describing characteristics of predatory journals. It is possible that further characteristics would have been included in our results if non-empirical research articles were not excluded. We chose to exclude these types of articles as they are more likely to be based on opinion or individual experience rather than evidence. Thirdly, our focus was on the biomedical literature. Whether the publication (e.g., having an IMRAD (Introduction Methods Results And Discussion) and peer review norms we’ve used apply across other disciplines is likely an important topic for further investigation. Fourthly, some of the studies included in our review are confounded by being identified through Beall’s lists, and journal publisher websites, which are considered controversial. Finally, we limited our study to English articles. It is possible that work published in other languages may have provided additional characteristics of predatory journals.

Reaching a consensus on what defines predatory journals, and what features reflect these, may be particularly useful to stakeholders (e.g., funders, research institutions) with a goal of establishing a list of vetted journals to recommend to their researchers. Such lists could be updated annually. Lists which attempt to curate predatory journals rather than legitimate journals are unlikely to achieve success given the reactive nature of this type of curation and the issue that new journals cannot easily be systematically discovered for evaluation ^25^. The development and use of digital technologies to provide information about journal publication practices (e.g., membership in the Committee on Publication Ethics ( https://publicationethics.org/), listing in the Directory of Open Access Journals ( https://doaj.org/)) may also prove to be a fruitful approach in reducing researchers’ submissions to predatory journals; empowering authors with knowledge is an important step in decision-making. Currently, researchers receive little education or support about navigating journal selection and submission processes. We envision a plug-in tool that researchers could click to get immediate feedback about a journal page they are visiting and whether it has characteristics of predatory journals. This feedback could provide them with the relevant information to determine if the journal suits their needs and/or meets any policy requirements to which they must adhere (e.g., digital preservation, indexing).

## Data availability

Study data and tables are available on the Open Science Framework, see: https://osf.io/4zm3t/.

Data are available under the terms of the Creative Commons Attribution 4.0 International license (CC-BY 4.0).

## Supplementary material

**Supplementary file 1:** Search Strategy

**Supplementary file 2:** Full citations of included articles.
