# Supplementary material for: What is a predatory journal? A scoping review
Source: F1000Res. 2018 Aug 23;7:1001. Originally published 2018 Jul 4. [Version 2] doi: 10.12688/f1000research.15256.2 (PMC6092896; doi:10.12688/f1000research.15256.2)
Supplement: Supplementary file 1 [file f1000research-7-17518-s0000.tgz › e70628d7-549e-4eb0-927a-db10444120d0_Supplemental_file_1_.docx]

**Supplementary File 1:** Search Strategy

Final Searches

2018 Jan 2

Database: Embase Classic+Embase <1947 to 2017 December 29>, Ovid MEDLINE(R) ALL <1946 to December 29, 2017>, PsycINFO <1806 to December Week 4 2017>, ERIC <1965 to November 2017>

Search Strategy:

--------------------------------------------------------------------------------

1 (predator* adj3 edit*).tw,kw,kf. (25)

2 (predator* adj3 journal*).tw,kw,kf. (307)

3 (predator* adj3 periodical?).tw,kw,kf. (6)

4 (predator* adj3 publication?).tw,kw,kf. (38)

5 (predator* adj3 publish*).tw,kw,kf. (304)

6 (bogus adj3 edit*).tw,kw,kf. (2)

7 (bogus adj3 journal*).tw,kw,kf. (6)

8 (bogus adj3 periodical?).tw,kw,kf. (0)

9 (bogus adj3 publication?).tw,kw,kf. (0)

10 (bogus adj3 publish*).tw,kw,kf. (1)

11 (decepti* adj3 edit*).tw,kw,kf. (21)

12 (decepti* adj3 journal*).tw,kw,kf. (16)

13 (decepti* adj3 periodical?).tw,kw,kf. (0)

14 (decepti* adj3 publication?).tw,kw,kf. (4)

15 (decepti* adj3 publish*).tw,kw,kf. (16)

16 (disreput* adj3 edit*).tw,kw,kf. (0)

17 (disreput* adj3 journal*).tw,kw,kf. (4)

18 (disreput* adj3 periodical?).tw,kw,kf. (0)

19 (disreput* adj3 publication?).tw,kw,kf. (3)

20 (disreput* adj3 publish*).tw,kw,kf. (0)

21 (distrust* adj3 edit*).tw,kw,kf. (1)

22 (distrust* adj3 journal*).tw,kw,kf. (2)

23 (distrust* adj3 periodical?).tw,kw,kf. (0)

24 (distrust* adj3 publication?).tw,kw,kf. (0)

25 (distrust* adj3 publish*).tw,kw,kf. (3)

26 (exploit* adj3 edit*).tw,kw,kf. (96)

27 (exploit* adj3 journal*).tw,kw,kf. (27)

28 (exploit* adj3 periodical?).tw,kw,kf. (1)

29 (exploit* adj3 publication?).tw,kw,kf. (38)

30 (exploit* adj3 publish*).tw,kw,kf. (95)

31 (fake? adj3 edit*).tw,kw,kf. (8)

32 (fake? adj3 journal*).tw,kw,kf. (29)

33 (fake? adj3 periodical?).tw,kw,kf. (0)

34 (fake? adj3 publication?).tw,kw,kf. (3)

35 (fake? adj3 publish*).tw,kw,kf. (15)

36 (hoax$2 adj3 edit*).tw,kw,kf. (1)

37 (hoax$2 adj3 journal*).tw,kw,kf. (5)

38 (hoax$2 adj3 periodical?).tw,kw,kf. (0)

39 (hoax$2 adj3 publication?).tw,kw,kf. (2)

40 (hoax$2 adj3 publish*).tw,kw,kf. (5)

41 (illegitim* adj3 edit*).tw,kw,kf. (3)

42 (illegitim* adj3 journal*).tw,kw,kf. (11)

43 (illegitim* adj3 periodical?).tw,kw,kf. (0)

44 (illegitim* adj3 publication?).tw,kw,kf. (7)

45 (illegitim* adj3 publish*).tw,kw,kf. (9)

46 (mislead* adj3 edit*).tw,kw,kf. (43)

47 (mislead* adj3 journal*).tw,kw,kf. (35)

48 (mislead* adj periodical?).tw,kw,kf. (0)

49 (mislead* adj3 publication?).tw,kw,kf. (58)

50 (mislead* adj publish*).tw,kw,kf. (5)

51 (non-legitim* adj3 edit*).tw,kw,kf. (0)

52 (non-legitim* adj3 journal*).tw,kw,kf. (0)

53 (non-legitim* adj3 periodical?).tw,kw,kf. (0)

54 (non-legitim* adj3 publication?).tw,kw,kf. (0)

55 (non-legitim* adj3 publish*).tw,kw,kf. (0)

56 (questionabl* adj3 edit*).tw,kw,kf. (23)

57 (questionabl* adj3 journal*).tw,kw,kf. (37)

58 (quesionabl* adj3 periodical?).tw,kw,kf. (0)

59 (questionabl* adj3 publication?).tw,kw,kf. (39)

60 (questionabl* adj3 publish*).tw,kw,kf. (50)

61 (racket? adj3 edit*).tw,kw,kf. (0)

62 (racket? adj3 journal*).tw,kw,kf. (1)

63 (racket? adj3 periodical?).tw,kw,kf. (0)

64 (racket? adj3 publication?).tw,kw,kf. (0)

65 (racket? adj3 publish*).tw,kw,kf. (0)

66 (scam* adj3 edit*).tw,kw,kf. (3)

67 (scam* adj3 journal*).tw,kw,kf. (10)

68 (scam* adj3 periodical?).tw,kw,kf. (0)

69 (scam* adj3 publication?).tw,kw,kf. (0)

70 (scam* adj3 publish*).tw,kw,kf. (2)

71 (sham adj3 edit*).tw,kw,kf. (0)

72 (sham adj3 journal*).tw,kw,kf. (7)

73 (sham adj3 periodical?).tw,kw,kf. (0)

74 (sham adj3 publication?).tw,kw,kf. (2)

75 (sham adj3 publish*).tw,kw,kf. (51)

76 (spam* adj3 edit*).tw,kw,kf. (1)

77 (spam* adj3 journal*).tw,kw,kf. (3)

78 (spam* adj3 periodical?).tw,kw,kf. (0)

79 (spam* adj3 publication?).tw,kw,kf. (1)

80 (spam* adj3 publish*).tw,kw,kf. (6)

81 (unethic* adj3 edit*).tw,kw,kf. (21)

82 (unethic* adj3 journal*).tw,kw,kf. (21)

83 (unethic* adj3 periodical?).tw,kw,kf. (0)

84 (unethic* adj3 publication?).tw,kw,kf. (48)

85 (unethic* adj3 publish*).tw,kw,kf. (51)

86 (unprofessional* adj3 edit*).tw,kw,kf. (1)

87 (unprofessional* adj3 journal*).tw,kw,kf. (3)

88 (unprofessional* adj3 periodical*).tw,kw,kf. (0)

89 (unprofessional* adj3 publication?).tw,kw,kf. (3)

90 (unprofessional* adj3 publish*).tw,kw,kf. (1)

91 (untrust* adj3 edit*).tw,kw,kf. (0)

92 (untrust* adj3 journal*).tw,kw,kf. (0)

93 (untrust* adj3 periodical?).tw,kw,kf. (0)

94 (untrust* adj3 publication?).tw,kw,kf. (1)

95 (untrust* adj3 publish*).tw,kw,kf. (2)

96 pseudo-journal*.tw,kw,kf. (10)

97 pseudo-periodical*.tw,kw,kf. (5)

98 pseudo-publish*.tw,kw,kf. (1)

99 Beall* list.tw,kw,kf. (40)

100 or/1-99 (1335)

101 limit 100 to yr="2012-current" (897)

102 remove duplicates from 101 (514) [TOTAL UNIQUE RECORDS]

103 102 use medall [MEDLINE UNIQUE RECORDS] (333)

104 102 use emczd [EMBASE RECORDS] (102)

105 102 use eric [ERIC UNIQUE RECORDS] (9)

106 102 not (103 or 104 or 105) [PSYCINFO UNIQUE RECORDS] (70)

***************************

Web of Science

| # 23 | [698](http://apps.webofknowledge.com.proxy.bib.uottawa.ca/summary.do?product=WOS&doc=1&qid=75&SID=8DB2hh2LbydCoiC1Dyr&search_mode=AdvancedSearch&update_back2search_link_param=yes) | #22  Indexes=SCI-EXPANDED, SSCI, A&HCI, CPCI-S, CPCI-SSH, ESCI Timespan=2012-2018 |
| --- | --- | --- |
| # 22 | [1,302](http://apps.webofknowledge.com.proxy.bib.uottawa.ca/summary.do?product=WOS&doc=1&qid=73&SID=8DB2hh2LbydCoiC1Dyr&search_mode=AdvancedSearch&update_back2search_link_param=yes) | #21 OR #20 OR #19 OR #18 OR #17 OR #16 OR #15 OR #14 OR #13 OR #12 OR #11 OR #10 OR #9 OR #8 OR #7 OR #6 OR #5 OR #4 OR #3 OR #2 OR #1  Indexes=SCI-EXPANDED, SSCI, A&HCI, CPCI-S, CPCI-SSH, ESCI Timespan=1900-2018 |
| # 21 | [20](http://apps.webofknowledge.com.proxy.bib.uottawa.ca/summary.do?product=WOS&doc=1&qid=72&SID=8DB2hh2LbydCoiC1Dyr&search_mode=AdvancedSearch&update_back2search_link_param=yes) | TS=(Beall* NEAR/1 list)  Indexes=SCI-EXPANDED, SSCI, A&HCI, CPCI-S, CPCI-SSH, ESCI Timespan=1900-2018 |
| # 20 | [27](http://apps.webofknowledge.com.proxy.bib.uottawa.ca/summary.do?product=WOS&doc=1&qid=71&SID=8DB2hh2LbydCoiC1Dyr&search_mode=AdvancedSearch&update_back2search_link_param=yes) | TS=(pseudo-journal* OR pseudo-periodical* OR pseudo-publish*)  Indexes=SCI-EXPANDED, SSCI, A&HCI, CPCI-S, CPCI-SSH, ESCI Timespan=1900-2018 |
| # 19 | [19](http://apps.webofknowledge.com.proxy.bib.uottawa.ca/summary.do?product=WOS&doc=1&qid=70&SID=8DB2hh2LbydCoiC1Dyr&search_mode=AdvancedSearch&update_back2search_link_param=yes) | TS=(untrust* NEAR/3 (edit* or journal* or periodical$ or publication$ or publish*))  Indexes=SCI-EXPANDED, SSCI, A&HCI, CPCI-S, CPCI-SSH, ESCI Timespan=1900-2018 |
| # 18 | [2](http://apps.webofknowledge.com.proxy.bib.uottawa.ca/summary.do?product=WOS&doc=1&qid=69&SID=8DB2hh2LbydCoiC1Dyr&search_mode=AdvancedSearch&update_back2search_link_param=yes) | TS=(unprofessional* NEAR/3 (edit* or journal* or periodical$ or publication$ or publish*))  Indexes=SCI-EXPANDED, SSCI, A&HCI, CPCI-S, CPCI-SSH, ESCI Timespan=1900-2018 |
| # 17 | [65](http://apps.webofknowledge.com.proxy.bib.uottawa.ca/summary.do?product=WOS&doc=1&qid=68&SID=8DB2hh2LbydCoiC1Dyr&search_mode=AdvancedSearch&update_back2search_link_param=yes) | TS=(unethic* NEAR/3 (edit* or journal* or periodical$ or publication$ or publish*))  Indexes=SCI-EXPANDED, SSCI, A&HCI, CPCI-S, CPCI-SSH, ESCI Timespan=1900-2018 |
| # 16 | [17](http://apps.webofknowledge.com.proxy.bib.uottawa.ca/summary.do?product=WOS&doc=1&qid=67&SID=8DB2hh2LbydCoiC1Dyr&search_mode=AdvancedSearch&update_back2search_link_param=yes) | TS=(spam* NEAR/3 (edit* or journal* or periodical$ or publication$ or publish*))  Indexes=SCI-EXPANDED, SSCI, A&HCI, CPCI-S, CPCI-SSH, ESCI Timespan=1900-2018 |
| # 15 | [68](http://apps.webofknowledge.com.proxy.bib.uottawa.ca/summary.do?product=WOS&doc=1&qid=66&SID=8DB2hh2LbydCoiC1Dyr&search_mode=AdvancedSearch&update_back2search_link_param=yes) | TS=(sham* NEAR/3 (edit* or journal* or periodical$ or publication$ or publish*))  Indexes=SCI-EXPANDED, SSCI, A&HCI, CPCI-S, CPCI-SSH, ESCI Timespan=1900-2018 |
| # 14 | [20](http://apps.webofknowledge.com.proxy.bib.uottawa.ca/summary.do?product=WOS&doc=1&qid=65&SID=8DB2hh2LbydCoiC1Dyr&search_mode=AdvancedSearch&update_back2search_link_param=yes) | TS=(scam* NEAR/3 (edit* or journal* or periodical$ or publication$ or publish*))  Indexes=SCI-EXPANDED, SSCI, A&HCI, CPCI-S, CPCI-SSH, ESCI Timespan=1900-2018 |
| # 13 | [2](http://apps.webofknowledge.com.proxy.bib.uottawa.ca/summary.do?product=WOS&doc=1&qid=64&SID=8DB2hh2LbydCoiC1Dyr&search_mode=AdvancedSearch&update_back2search_link_param=yes) | TS=(racket$ NEAR/3 (edit* or journal* or periodical$ or publication$ or publish*))  Indexes=SCI-EXPANDED, SSCI, A&HCI, CPCI-S, CPCI-SSH, ESCI Timespan=1900-2018 |
| # 12 | [120](http://apps.webofknowledge.com.proxy.bib.uottawa.ca/summary.do?product=WOS&doc=1&qid=63&SID=8DB2hh2LbydCoiC1Dyr&search_mode=AdvancedSearch&update_back2search_link_param=yes) | TS=(questionabl* NEAR/3 (edit* or journal* or periodical$ or publication$ or publish*))  Indexes=SCI-EXPANDED, SSCI, A&HCI, CPCI-S, CPCI-SSH, ESCI Timespan=1900-2018 |
| # 11 | [1](http://apps.webofknowledge.com.proxy.bib.uottawa.ca/summary.do?product=WOS&doc=1&qid=62&SID=8DB2hh2LbydCoiC1Dyr&search_mode=AdvancedSearch&update_back2search_link_param=yes) | TS=(non-legitim* NEAR/3 (edit* or journal* or periodical$ or publication$ or publish*))  Indexes=SCI-EXPANDED, SSCI, A&HCI, CPCI-S, CPCI-SSH, ESCI Timespan=1900-2018 |
| # 10 | [140](http://apps.webofknowledge.com.proxy.bib.uottawa.ca/summary.do?product=WOS&doc=1&qid=61&SID=8DB2hh2LbydCoiC1Dyr&search_mode=AdvancedSearch&update_back2search_link_param=yes) | TS=(mislead* NEAR/3 (edit* or journal* or periodical$ or publication$ or publish*))  Indexes=SCI-EXPANDED, SSCI, A&HCI, CPCI-S, CPCI-SSH, ESCI Timespan=1900-2018 |
| # 9 | [28](http://apps.webofknowledge.com.proxy.bib.uottawa.ca/summary.do?product=WOS&doc=1&qid=60&SID=8DB2hh2LbydCoiC1Dyr&search_mode=AdvancedSearch&update_back2search_link_param=yes) | TS=(illegitim* NEAR/3 (edit* or journal* or periodical$ or publication$ or publish*))  Indexes=SCI-EXPANDED, SSCI, A&HCI, CPCI-S, CPCI-SSH, ESCI Timespan=1900-2018 |
| # 8 | [15](http://apps.webofknowledge.com.proxy.bib.uottawa.ca/summary.do?product=WOS&doc=1&qid=59&SID=8DB2hh2LbydCoiC1Dyr&search_mode=AdvancedSearch&update_back2search_link_param=yes) | TS=(hoax* NEAR/3 (edit* or journal* or periodical$ or publication$ or publish*))  Indexes=SCI-EXPANDED, SSCI, A&HCI, CPCI-S, CPCI-SSH, ESCI Timespan=1900-2018 |
| # 7 | [51](http://apps.webofknowledge.com.proxy.bib.uottawa.ca/summary.do?product=WOS&doc=1&qid=58&SID=8DB2hh2LbydCoiC1Dyr&search_mode=AdvancedSearch&update_back2search_link_param=yes) | TS=(fake$ NEAR/3 (edit* or journal* or periodical$ or publication$ or publish*))  Indexes=SCI-EXPANDED, SSCI, A&HCI, CPCI-S, CPCI-SSH, ESCI Timespan=1900-2018 |
| # 6 | [387](http://apps.webofknowledge.com.proxy.bib.uottawa.ca/summary.do?product=WOS&doc=1&qid=57&SID=8DB2hh2LbydCoiC1Dyr&search_mode=AdvancedSearch&update_back2search_link_param=yes) | TS=(exploit* NEAR/3 (edit* or journal* or periodical$ or publication$ or publish*))  Indexes=SCI-EXPANDED, SSCI, A&HCI, CPCI-S, CPCI-SSH, ESCI Timespan=1900-2018 |
| # 5 | [11](http://apps.webofknowledge.com.proxy.bib.uottawa.ca/summary.do?product=WOS&doc=1&qid=56&SID=8DB2hh2LbydCoiC1Dyr&search_mode=AdvancedSearch&update_back2search_link_param=yes) | TS=(distrust* NEAR/3 (edit* or journal* or periodical$ or publication$ or publish*))  Indexes=SCI-EXPANDED, SSCI, A&HCI, CPCI-S, CPCI-SSH, ESCI Timespan=1900-2018 |
| # 4 | [8](http://apps.webofknowledge.com.proxy.bib.uottawa.ca/summary.do?product=WOS&doc=1&qid=55&SID=8DB2hh2LbydCoiC1Dyr&search_mode=AdvancedSearch&update_back2search_link_param=yes) | TS=(disreput* NEAR/3 (edit* or journal* or periodical$ or publication$ or publish*))  Indexes=SCI-EXPANDED, SSCI, A&HCI, CPCI-S, CPCI-SSH, ESCI Timespan=1900-2018 |
| # 3 | [43](http://apps.webofknowledge.com.proxy.bib.uottawa.ca/summary.do?product=WOS&doc=1&qid=54&SID=8DB2hh2LbydCoiC1Dyr&search_mode=AdvancedSearch&update_back2search_link_param=yes) | TS=(decepti* NEAR/3 (edit* or journal* or periodical$ or publication$ or publish*))  Indexes=SCI-EXPANDED, SSCI, A&HCI, CPCI-S, CPCI-SSH, ESCI Timespan=1900-2018 |
| # 2 | [6](http://apps.webofknowledge.com.proxy.bib.uottawa.ca/summary.do?product=WOS&doc=1&qid=53&SID=8DB2hh2LbydCoiC1Dyr&search_mode=AdvancedSearch&update_back2search_link_param=yes) | TS=(bogus NEAR/3 (edit* or journal* or periodical$ or publication$ or publish*))  Indexes=SCI-EXPANDED, SSCI, A&HCI, CPCI-S, CPCI-SSH, ESCI Timespan=1900-2018 |
| # 1 | [336](http://apps.webofknowledge.com.proxy.bib.uottawa.ca/summary.do?product=WOS&doc=1&qid=52&SID=8DB2hh2LbydCoiC1Dyr&search_mode=AdvancedSearch&update_back2search_link_param=yes) | TS=(predator* NEAR/3 (edit* or journal* or periodical$ or publication$ or publish*))  Indexes=SCI-EXPANDED, SSCI, A&HCI, CPCI-S, CPCI-SSH, ESCI Timespan=1900-2018 |
