# Supplementary material for: What is a predatory journal? A scoping review
Source: F1000Res. 2018 Aug 23;7:1001. Originally published 2018 Jul 4. [Version 2] doi: 10.12688/f1000research.15256.2 (PMC6092896; doi:10.12688/f1000research.15256.2)
Supplement: Supplementary file 2 [file f1000research-7-17518-s0001.tgz › bdaf8374-b326-4432-9f98-30e3444e691c.docx]

**Appendix 2.** Full citations of included articles

1. [RefID: 1]. Oermann MH, Nicoll LH, Chinn PL, et al. Quality of articles published in predatory nursing journals. Nurs Outlook 2018 Jan;66(1):4-10.

2. [RefID: 8]. McCann TV, Polacsek M. False gold: Safely navigating open access publishing to avoid predatory publishers and journals. J Adv Nurs 2018 Apr;74(4):809-17.

3. [RefID: 13]. Mercier E, Tardif PA, Moore L, et al. Invitations received from potential predatory publishers and fraudulent conferences: a 12-month early-career researcher experience. Postgrad Med J 2018 Feb;94(1108):104-8.

4. [RefID: 35]. Bolshete P. Analysis of thirteen predatory publishers: a trap for eager-to-publish researchers. Curr Med Res Opin 2018;34(1):157-62.

5. [RefID: 99]. Manca A, Martinez G, Cugusi L, et al. The surge of predatory open-access in neurosciences and neurology. Neuroscience 2017;353:166-73.

6. [RefID: 121]. Christopher MM, Young KM. Awareness of "Predatory" Open-Access Journals among Prospective Veterinary and Medical Authors Attending Scientific Writing Workshops. Front vet sci 2015;2:22.

7. [RefID: 150]. Oermann MH, Conklin JL, Nicoll LH, et al. Study of Predatory Open Access Nursing Journals. J Nurs Scholarsh 2016;48(6):624-32.

8. [RefID: 165]. Sorokowski P, Kulczycki E, Sorokowska A, et al. Predatory journals recruit fake editor. Nature 2017;543(7646):481-3.

9. [RefID: 168]. Manca A, Martinez G, Cugusi L, et al. Predatory Open Access in Rehabilitation. Arch Phys Med Rehabil 2017;98(5):1051-6.

10. [RefID: 176]. Hansoti B, Langdorf MI, Murphy LS. Discriminating Between Legitimate and Predatory Open Access Journals: Report from the International Federation for Emergency Medicine Research Committee. West J Emerg Med 2016;17(5):497-507.

11. [RefID: 181]. Bugeja J, Grech V. Email solicitation for scholarly work--a single researcher's perspective. J Vis Commun Med 2015;38(3-4):231-3.

12. [RefID: 203]. Wicherts JM. Peer Review Quality and Transparency of the Peer-Review Process in Open Access and Subscription Journals. PloS one 2016;11(1):e0147913.

13. [RefID: 209]. Shen C, Bjork BC. 'Predatory' open access: a longitudinal study of article volumes and market characteristics. BMC Med 2015;13:230.

14. [RefID: 275]. Djuric D. Penetrating the omerta of predatory publishing: the romanian connection. Sci Eng Ethics 2015;21(1):183-202.

15. [RefID: 299]. Shamseer L, Moher D, Maduekwe O, et al. Potential predatory and legitimate biomedical journals: can you tell the difference? A cross-sectional comparison. BMC Med 2017;15(1):28.

16. [RefID: 362]. Clemons M, De Costa E Silva, Joy AA, et al. Predatory invitations from journals: More than just a nuisance? Oncologist 2017;22(2):236-40.

17. [RefID: 384]. Moher D, Srivastava A. You are invited to submit. BMC Med 2015;13(1):180.

18. [RefID: 462]. McCutcheon LE, Aruguete MS, McKelvie SJ, et al. How questionable are predatory social science journals? North American Journal of Psychology 2016;18(3):427-40.

19. [RefID: 489]. No ai. Predatory publishing companies: Ethical considerations in the open-access era. Journal of Developmental and Behavioral Pediatrics 2015;36(3):223-4.

20. [RefID: 525]. Frandsen TF. Are predatory journals undermining the credibility of science? A bibliometric analysis of citers. Scientometrics 2017;113(3):1513-28.

21. [RefID: 548]. da Silva JAT. Copycats and impostors in science publishing: the case of Current Science. Current Science 2017;113(5):834.

22. [RefID: 561]. de Jager P, de Kock F, van der Spuy P. Do not feed the predators. South African Journal of Business Management 2017;48(3):35-45.

23. [RefID: 586]. Noga-Styron KE, Olivero JM, Britto S. Predatory Journals in the Criminal Justices Sciences: Getting our Cite on the Target. Journal of Criminal Justice Education 2017;28(2):174-91.

24. [RefID: 596]. Mccool JH. Why I Published in a Predatory Journal. Scientist 2017;31(6):23.

25. [RefID: 654]. Ibba S, Pani FE, Stockton JG, et al. Incidence of predatory journals in computer science literature. Library Review 2017;66(6-7):505-22.

26. [RefID: 660]. Cosentino M, Marino F, Haldar C, et al. Honorary Authors and Unwitting: Personal Experience with A Journal from A Publisher Included in the Beall'S List of Predatory Publishers. Plagiarism Across Europe and Beyond 2017 2017;15-24.

27. [RefID: 686]. Marchitelli A, Galimberti P, Bollini A, et al. Improvement of editorial quality of journals indexed in DOAJ: a data analysis. Jlis It 2017;8(1):1-21.

28. [RefID: 701]. Seethapathy GS, Kumar JUS, Hareesha AS. India's scientific publication in predatory journals: need for regulating quality of Indian science and education. Current Science 2016;111(11):1759-64.

29. [RefID: 728]. Martin A, Martin T. A not-so-harmless experiment in predatory open access publishing. Learned Publishing 2016;29(4):301-5.

30. [RefID: 736]. Somoza-Fernandez M, Rodriguez-Gairin JM, Urbano C. Presence of Alleged Predatory Journals in Bibliographic Databases: Analysis of Beall'S List. Profesional de la Informacion 2016;25(5):730-7.

31. [RefID: 755]. Kozak M, Iefremova O, Hartley J. Spamming in Scholarly Publishing: A Case Study. Journal of the Association for Information Science and Technology 2016;67(8):2009-15.

32. [RefID: 812]. Petrisor AI. Evolving strategies of the predatory journals. Malaysian Journal of Library & Information Science 2016;21(1):1-17.

33. [RefID: 900]. Xia JF, Harmon JL, Connolly KG, et al. Who publishes in "predatory" journals? Journal of the Association for Information Science and Technology 2015;66(7):1406-17.

34. [RefID: 904]. Jalalian M, Dadkhah M. The full story of 90 hijacked journals from August 2011 to June 2015. Geographica Pannonica 2015;19(2):73-87.

35. [RefID: 975]. Ezinwa Nwagwu W, Ojemeni O. Penetration of Nigerian predatory biomedical open access journals 2007-2012: a bibiliometric study. Learned Publishing 2015;28(1):23-34.

36. [RefID: 976]. Xia JF. Predatory journals and their article publishing charges. Learned Publishing 2015;28(1):69-74.

37. [RefID: 1012]. Omobowale AO, Akanle O, Adeniran AI, et al. Peripheral scholarship and the context of foreign paid publishing in Nigeria. Current Sociology 2014;62(5):666-84.

38. [RedID: 1068]:Markowitz DM, Powell JH, Hancock JT. The Writing Style of Predatory Publishers. 2014.
