# Supplementary material for: What is a predatory journal? A scoping review
Source: F1000Res. 2018 Aug 23;7:1001. Originally published 2018 Jul 4. [Version 2] doi: 10.12688/f1000research.15256.2 (PMC6092896; doi:10.12688/f1000research.15256.2)
Supplement: Peer review history

# Peer review: Referee response for version 2

**DOI:** 10.5256/f1000research.17518.r37491

**Published:** 2018-09-13

**Authors:** Johann Mouton

**Affiliations:** 1 Centre for Research on Evaluation, Science and Technology (CREST), DST/NRF Centre of Excellence in Scientometrics and Science, Technology and Innovation Policy, Stellenbosch University, Stellenbosch, South Africa

It is clear from the response of the authors that they disagree that my criticism is relevant. And therefore they have chosen not to change their paper to accommodate these. In fairness this would have required a substantial rewrite.  I believe my comments remain appropriate. However, I have also taken into account the more positive responses from the other reviewers. I have hence decided to agree that the paper can be indexed - even though it makes less of a contribution than what I would have liked.

I have read this submission. I believe that I have an appropriate level of expertise to confirm that it is of an acceptable scientific standard.

# Peer review: Referee response for version 1

**DOI:** 10.5256/f1000research.16618.r36291

**Published:** 2018-08-13

**Authors:** Joanna Chataway

**Affiliations:** 1 SPRU—Science Policy Research Unit, University of Sussex, Brighton, UK

This is an interesting and very useful article on a subject which, as the authors note, is widely discussed but under researched. 

 The article sets out to examine data derived from a scoping review  in an effort to contribute to a definition of the term 'predatory journal'.  Disagreements about whether the term should be used at all are summarised early on in the article and this provides a useful backdrop to the multiple difficulties involved in defining the term. 

**Questions and issues raised by the article**

 In considering the issue of predatory journals, the authors raise questions of what might be considered characteristics of a legitimate journal might.  The article discusses this only partially and mainly in relation to the difficulties of distinguishing between journals which set out to mislead and which abandon the aims of publish high quality science entirely and, on the other hand, those which are poorly managed and run. It is not necessary I think to give this further and detailed consideration here, but it is important to note that there may well other issues to consider here.  For example, there may well be complex relationships between the practices of legitimate journals, and the unintended consequences of impact factor metrics (as noted in The Lancet special issue on 'Increasing value, reducing waste' cited by the authors for example) and the expansion of bad as well as good journals and publication platforms which offer alternatives. The Lancet and other critiques point to  intense competition involved in publishing in high impact journals, the need to publish for promotion and employment and so on as factors which drive bad practice in general and may also play a role in the rise of predatory journals.

 Another issue which is only briefly mentioned in the article is whether the norms of publishing and peer review differ across different disciplines. Perhaps give the characteristics of existing literature it is not possible to say much about this currently, but the authors could raise more clearly this as an issue to be considered in future research.  And I think the point should be made that whilst it is common for health research articles to follow the reporting convention of 'Introduction, methods, results, discussion', this is not the case in other fields. Thus having this as a criteria for judging the quality of a journal could be misleading. 

**Clarification of terminology**

 I would encourage the authors to explain terms such as 'epidemiological characteristics' and 'scoping review' which may be familiar to those who work in health research but not perhaps to others. 

**Some examples?**

 Some of the results would have been clearer to me if examples had been included.  This is particularly the case with regard to 'persuasive language'.  It is unclear to me what is being referred to by that term.  

**Missing link?**

 I couldn't get the link to further details about the search strategy to work.  That accounts for the 'partial' score for source data question but that may just be a problem for me and not for others.

I have read this submission. I believe that I have an appropriate level of expertise to confirm that it is of an acceptable scientific standard.

# Response

**Published:** 2018-08-18

**Authors:** David Moher

**Affiliations:** Ottawa Hospital Research Institute, Canada

In response to Joanna Chataway’s review:

- We have made some modifications to the limitations section of our paper. We now state (version 2) “Thirdly, our focus was on the biomedical literature. Whether the publication (e.g., having an IMRAD (Introduction Methods Results And Discussion) and peer review norms we’ve used apply across other disciplines is likely an important topic for further investigation.”
- We have more cleared described scoping reviews (version 2).
- We believe we have given some examples in Table 3. For example, in response to the query as to the use and meaning of “persuasive language”, we state (Version 1 and version 2) “Language that targets; Language that attempts to convince the author to do or believe something”.
- We have fixed the broken link to the full search strategy (version 2).

# Peer review: Referee response for version 1

**DOI:** 10.5256/f1000research.16618.r36292

**Published:** 2018-08-13

**Authors:** Johann Mouton

**Affiliations:** 1 Centre for Research on Evaluation, Science and Technology (CREST), DST/NRF Centre of Excellence in Scientometrics and Science, Technology and Innovation Policy, Stellenbosch University, Stellenbosch, South Africa

The paper ultimately promises more than what it delivers. It presents the results of an analysis which has resulted in a set of characteristics of predatory journals derived from a scoping review of recent studies. However, the final discussion section is extremely disappointing. There is no attempt by the authors to add much value to the rather fragmented results found through the review. Part of the problem is that the characteristics listed are treated as equally weighted.  Most of the authors who have written on the phenomenon of predatory journals in recent years have attempted to end up with a set of fairly authoritative and even 'objective' criteria that would by themselves be sufficient to classify a journal as predatory. Some of these characteristics would include referencing fake indexing, fake impact metrics, not being indexed in the DOAJ's and a few more.  In order to get to a 'consensus' view of what are the key characteristics of a predatory journal, a simple listing of all possible characteristics will not take us much further. 

 It is perhaps then not surprising that their recommendation is for a consensus type meeting where experts could work towards a consensus definition.

 More to the point: in my view to get to the kind of end goal of a consensus or more widely acceptable definition, would require a more theoretical or at least conceptual framework that is embedded in some of the work on scientific communication and publishing which stipulates what good practices in (journal) publishing are.

 Unfortunately this paper does not help us much on the way to this goal.

I have read this submission. I believe that I have an appropriate level of expertise to state that I do not consider it to be of an acceptable scientific standard, for reasons outlined above.

# Response

**Published:** 2018-08-18

**Authors:** David Moher

**Affiliations:** Ottawa Hospital Research Institute, Canada

We are sorry to disappoint Johann Mouton in our scoping review. We believe that a scoping review is a reasonable way to attempt to map the literature. Scoping reviews do not typically weight included studies. We believe our review highlights some of the disagreements in the literature about presumed relevant characteristics of presumed predatory journals.

# Peer review: Referee response for version 1

**DOI:** 10.5256/f1000research.16618.r35740

**Published:** 2018-08-01

**Authors:** Monica Berger

**Affiliations:** 1 CUNY New York City College of Technology, Brooklyn, NY, USA

This article represents a unique contribution to what has been written on this topic. 

 Although the core readership for F1000Research consists of scientists, I am sure this article will be read by many non-scientists and many of my suggestions to the authors relate to this point.

 A general comment: the authors should note that most of the data used for empirical studies of predatory publishing is drawn from Beall's List and Beall's List was and is still controversial [the authors' discussion of Beall's List under **Introduction** is balanced and articulate]. Essentially, any empirical study of predatory publishing is based on one or two sources of data: Beall's List and/or email solicitations which lead to journals and their publisher websites. This should be made explicit.

 The data that underlies much of the literature is very fuzzy and subjective. Without cross-checking publisher and journal data (e.g. many predatory publishers claim inclusion in DOAJ and this data point is particularly sticky), and probing the content, the underlying literature is limited. Moher, David et al's ^1^ study seems to be one of the only studies to examine content and evaluates the methodological design and research protocols of articles but, as the authors note, it gets excluded because of its publication in commentary format!

 The overall quantification of the literature differentiating empirical vs. editorial is extremely helpful.

 I found the raw data of characteristics pretty overwhelming and I wonder if the authors could somehow aggregate or otherwise organize the information in a way that makes it easier to scan. I recognize that they have summarized their data in the body of the article.

 The conclusions clearly address the limitations of the study but what I think would be most important is teasing out where the data came from: publisher emails leading to publisher websites, and/or Beall's List leading to journals and publisher websites. Both are imperfect sources.

 I would like to see this data used again with more aggregation. I recognize that scoping reviews are meant to be fast so this article's data could be used for further research.

 Specific comments:

**Introduction: **Agreed that some journals from the Global South provide important regional research but the authors should note that many of these Global South journals market themselves as "international" or "global" and do *not *focus on regional research because of a desire to cast a wide net. Legitimate, amateurish journals deemed as predatory from this group actually would be more likely to have a scope that is regional and specific as opposed to the multidisciplinary scope of many predatory publishers.

 The authors should explain far more explicitly what a scoping review is and its purpose. *Non-biomedical readers will be unfamiliar with this type of methodology/article.*

 I also am not entirely sure about the use of the word "epidemiological" in terms of discussing the topic at hand: non-biomedical readers may be unsure what exactly is meant.

 Lastly, as much as it is very helpful to identify characteristics of predatory journals as drawn from the literature, it seems somewhat positivist to use this very limited body of literature which is by its heavy use of Beall's List data as a means to "generate a consensus definition of predatory journals." Until there is more qualitative research and more multidisciplinary and longitudinal research as was done by Shen and Bjork, there are lacunae in the research literature. The recent articles based on the research by this team is, groundbreaking but largely limits scope to biomedical literature.

**Screening and data extraction**

 The use of implicit and explicit definitions is very important and valuable.

**Search strategy**

 It is possible that some research from librarians and information science scholars might have been missed. There is also some concern that if the articles are open access, they may have not been indexed in traditional databases. This concern relates to the **Data Analysis **section as well since *newer and smaller* open access journals may not have a Journal Impact Factor and be excluded from SCIMAGO.

**Mapping the data into emergent themes**

 Under the descriptor "persuasive language," the language of predatory journals targets authors and not readers. This should be explained.

 What is somewhat confusing to me is separating characteristics in the literature based on the authors' perceptions or evaluations of the journals and publishers versus the actual data drawn from the journals and publishers' emails, journals, articles, and websites. Whether or not the author of the underlying articles performed cross-checking is also important.

**Table 4 Characteristics**

 The characteristic **JOURNALS HAVE SHORT PEER REVIEW TIMES **isn't mapped to a descriptor but it is a very most important common characteristic of publisher appeals to authors. It typically maps to Poor Quality Standards although not in an absolute manner since obviously large, quality journals can also have quick turnaround. It is unclear to me if because this characteristic lacks a descriptor, it may lose weight in the analysis. I note that **JOURNALS HAVE SHORT/RAPID PUBLICATION TIMES **is also a NA descriptor. These two facets are closely related.

**ARTICLE SUBMISSION OCCURS VIA EMAIL **this may be a signal of poor standards but is often more a reflection of low budgets and the many amateurish journals that have been lumped into Beall's List.

**JOURNALS DO NOT CONTAIN ANY ARTICLES **the high number of predatory journals without articles is a very important data point that should be emphasized.

I have read this submission. I believe that I have an appropriate level of expertise to confirm that it is of an acceptable scientific standard.

## References

[1] MoherDShamseerLCobeyKLaluMGalipeauJAveyMAhmadzaiNAlabousiMBarbeauPBeckADanielRFrankRGhannadMHamelCHersiMHuttonBIsupovIMcGrathTMcInnesMPageMPrattMPussegodaKSheaBSrivastavaAStevensAThavornKvan KatwykSWardRWolfeDYazdiFYuAZiaiH: Stop this waste of people, animals and money. *Nature*.2017;549(7670) : 10.1038/549023a 23-25 10.1038/549023a 28880300

# Response

**Published:** 2018-08-11

**Authors:** David Moher

**Affiliations:** Ottawa Hospital Research Institute, Canada

We thank Monica Berger for her thoughtful peer review of our manuscript. We have made revisions throughout (version 2):

- We have further indicated the limitations of Beall’s lists for this type of research;
- We have noted the global south issue of journals using “international” or “global” in their titles; we have provided some clarity as to scoping reviews; i
- It is possible we’ve missed some relevant literature from our review (as is the potential in any review exercise) although we believe in its current form it is both broad and multidisciplinary. As a follow-up exercise we will reach out to library/expert listservs related to this field of enquiry;
- We agree that the two statements without a descriptor are important, however, the length of time for a peer-review or publication cannot be classified as either a positive or negative statement and hence were not given a descriptor term. While it could be mapped to Poor Quality Standards, we cannot assume that a short peer-review time is indicative of poor quality.

# Response

**Published:** 2018-08-11

**Authors:** David Moher

**Affiliations:** Ottawa Hospital Research Institute, Canada

In response to Valerie Ann Matarese’s comment we have changed two words in the introduction (version 2).

 Ross Mounce is misinformed. This is not a “literature review of opinion, and as such, one wonders what the value of the exercise is.”. As stated in the screening and data extortion section of the Methods of this scoping review (version 1) “we restricted our sample of articles to those that provided a definition of predatory journals, or described characteristics of predatory journals, based on empirical work (i.e., not opinion, not definitions which referenced previous work).”.

 We thank Edgardo Rolla for his comments on our scoping review (version 1).
